# Effect of ranolazine on plasma arginine derivatives and urinary isoprostane 8-iso-PGF_2α_ in patients with myocardial infarction in the randomized RIMINI-Trial

**DOI:** 10.1038/s41598-019-42239-1

**Published:** 2019-04-05

**Authors:** Tjark F. Schwemer, Navina Deutscher, Nadine Diermann, Rainer Böger, Edzard Schwedhelm, Stefan Blankenberg, Felix W. Friedrich

**Affiliations:** 1 Institute of Experimental Pharmacology and Toxicology, University Medical Center Hamburg-Eppendorf, Hamburg, Germany; 2 Cardiovascular Research Center, University Medical Center Hamburg-Eppendorf, Hamburg, Germany; 3 DZHK (German Centre for Cardiovascular Research), partner site Hamburg/Kiel/Lübeck, Hamburg, Germany; 4 University Heart Center Hamburg, Hamburg, Germany; 5 Institute of Clinical Pharmacology and Toxicology, University Medical Center Hamburg-Eppendorf, Hamburg, Germany

## Abstract

The purpose of the present study was to assess whether 6-week ranolazine application on top of guideline-based treatment impacts on the arginine/NO pathway and urinary isoprostane 8-iso-PGF_2α_ as marker of oxidative stress in patients directly after a myocardial infarction. 20 patients with unstable angina pectoris and proof of acute cardiac ischemia entered the study. 10 subjects received the study drug ranolazine in addition to standard treatment, the others received only standard treatment. Urine and venous blood were collected before and after treatment. At the end of the study and compared to baseline, homoarginine levels had increased in the control group. This was not the case in ranolazine-patients. Interestingly, in ranolazine-treated-patients arginine plasma levels were significantly higher at the end of the study than at baseline (difference +26 µmol/L, 95% CI 8.6 to 44 µmol/L). ADMA and SDMA levels were not different. Urine levels of the oxidative stress marker 8-iso-PGF_2α_ tended to be lower in ranolazine-treated patients (−144 pmol/mg creatinine). Findings of this hypothesis-driven study give evidence that ranolazine treatment enhances arginine plasma levels and lowers oxidative stress.

## Introduction

Coronary artery disease (CAD) is connected to high mortality and morbidity^1^. It can present as chronic stable angina and acute coronary syndrome (ACS). Present pharmacological ACS treatment consists of antiplatelet, beta-adrenoceptor and calcium channel antagonist, nitrate and high dose statin therapy^2^. Nitrates exert their effect by enhancing the oxygen supply/demand mismatch. They predominantly dilate veins, which decreases preload, lowering ventricular wall stress and myocardial oxygen demand. This improvement in subendocardial perfusion^2^ counteracts oxidative damage^3,4^. Nitrates are endothelium-independent vasodilatory drugs which by forming NO mimic the effects of endogenous NO on vascular smooth muscle. NO in turn activates the enzyme guanylyl cyclase to produce cGMP. This stimulates protein kinase G leading to dephosphorylation of the myosin light chain resulting in smooth muscle relaxation^5,6^. NO pathway dysfunction has been associated with CAD risk factors^7–9^. NO bioavailability is dependent on the efficient generation from its precursor arginine by endothelial nitric oxide synthase (eNOS), which can show uncoupling under conditions of oxidative stress^10^. Oral arginine supplementation has been controversially discussed since studies in CAD patients have shown positive and negative results^11–14^. NO synthesis can be decreased by asymmetric (ADMA) and symmetric dimethylarginine (SDMA), two methylation products of arginine protein residues by protein arginine methyltransferase 1 (PRMT-1) and PRMT-2^15,16^. High ADMA plasma levels have been linked to cardiovascular events^17,18^. Furthermore, low plasma homoarginine, an Arg homolog, which improves arginine availability, was identified as a risk marker for major adverse cardiovascular events in patients with acute chest pain^19^.

Ischemic myocardium displays an increase in the late Na^+^-current. This can deteriorate left ventricular function and dispose to arrhythmias via Ca^2+^ overload^20^. Recently published data showed that ranolazine, a late Na^+^-current inhibitor, improves myocardial blood flow and therefore microcirculation in the myocardium by reducing diastolic wall tension via inhibition of the late Na^+^-influx and consecutive Ca^2+^-overload in stable CAD patients^21^. Ranolazine has been approved for CAD treatment. We recently showed in a preliminary hypothesis-driven study (NCT01797484, ClinicalTrials.gov) that six-week ranolazine therapy decreased the area of dyskinetic myocardium in patients with ACS by trend^22^. Previous animal studies have shown an additional vasodilatory effect of ranolazine in aortic rings^23^. Precontracted rat aortic rings showed a concentration-dependent vasodilation in the presence of ranolazine, which could be reduced by inhibition of NO synthase. This indicates a connection between ranolazine and the NO pathway. The intent of the present study was to evaluate whether the application of ranolazine on top of the guideline-based treatment in ACS patients directly after a myocardial infarction has an impact on the arginine/NO pathway and oxidative stress marker urinary isoprostane 8-iso-PGF_2α_ since previous studies have shown that the 15-F_2trans_-isoprostane (15-F_2t_-IsoP, 8-iso-PGF_2α_, iPF_2α_-III) may serve as a valid marker for oxidative stress and therefore also a reliable marker of CAD^24–27^.

## Methods

### Patients and study protocol

For study details please refer to^22^ and the Supplemental File. In short, the study was performed in a two-armed, controlled, and randomized way. 10 patients received ranolazine additional to guideline-based standard treatment orally for 6 weeks (first seven days 500 mg bidaily, the next 35 days 750 mg ranolazine bidaily), whereas the 10 control patients received only standard ACS treatment. Urine and venous blood were collected before application of ranolazine and after 6 weeks of treatment. Urine was acidified between pH 2 and 4 and frozen at −80 °C in an aliquot of 15 ml until analysis. After centrifugation of blood samples, EDTA plasma aliquots were stored at −80 °C. Laboratory staff was blinded regarding specimen of study groups. We evaluated eligibility and obtained written informed consent as documented in the study protocol approved by the local Review Board for Studies in Humans, Hamburg. The study was executed in accordance to the principles of the Declaration of Helsinki (revised in Tokyo 1975, Venice 1983, Hong Kong 1989, Sommerset West 1996) and the ICH-based GCP Rules.

### Measurement of plasma arginine derivatives and urinary isoprostane 8-iso-PGF_2α_

Plasma arginine, homoarginine, ADMA and SDMA were determined from frozen EDTA plasma samples with a high throughput mass spectrometric (MS) assay, applying electrospray ionization/liquid chromatography (LC)-MS/MS^28–30^. In short, proteins were precipitated by 25 µL EDTA plasma to 100 µL of internal standards (stable isotope labelled arginine, ADMA, and homoarginine) dissolved in methanol, then centrifuged, evaporated, and afterwards transformed to their butyl ester derivatives using 1 N of butanolic hydrochloric acid. After a centrifugation step, eluates were dried by heating and redissolved in 100 µL methanol/water (25:75) with 0.1% ammonium formate before measurements were performed. Samples were transferred to a CTC PAL autosampler, and 20-µL aliquots were exposed to further MS system analysis (Varian 1200 MS; Agilent Technologies, Santa Clara, CA). The lower limits of quantification for arginine, ADMA, and homoarginine were 0.25, 0.005, and 0.1 µmol/L, respectively. All intra- and interassay coefficients of variation were ≤7.5%.

Urinary 8-iso-PGF_2α_ was purified by immunoaffinity chromatography and then measured by gas chromatography–mass spectrometry (GC-MS) as previously described^31^. Briefly, urinary samples (stored at −80 °C) were thawed, and the labelled internal standard ^2^H_4_-8-iso-PGF_2α_ was added at a concentration of 1 ng/ml. Afterwards the samples were sent through immunoaffinity columns (Cayman Chemicals, Ann Arbor, Michigan, USA) and derivatized as described before to attain the pentafluorobenzyl ester and trimethylsilyl ether derivatives^32^. 8-iso-PGF2α was identified at an m/z ratio of 569.4 and the internal standard ^2^H_4_-8-iso-PGF_2α_ at an m/z ratio of 573.4. Final results were expressed as pg of 8-iso-PGF_2α_/mg urinary creatinine.

### Statistical analysis

Data are given as mean ± SD and 95% confidence intervals (CI) or number and %. Comparisons were performed by paired (baseline vs. study end) or unpaired (standard vs. ranolazine) Student’s t-test, two-sided, using GraphPad Prism 6. A value of p < 0.05 was considered statistically significant.

## Results

**Figure 1:**
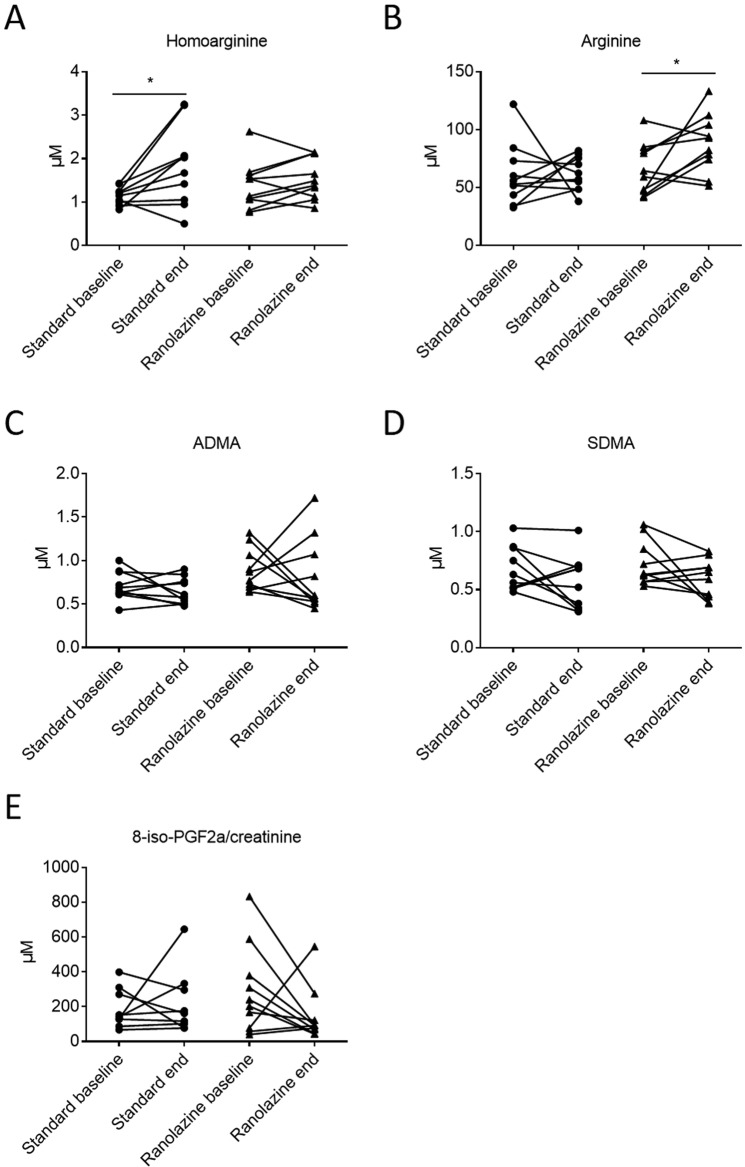
Plasma levels of NO pathway markers and urinary isoprostane 8-iso-PGF_2a_ at baseline and end of the study: Data are given as individual values at baseline and end of the study of (**A**) homoarginine, (**B**) arginine, (**C**) ADMA, (**D**) SDMA, (**E**) 8-iso-PGF_2a_/creatinine values. Comparisons were performed by paired (baseline vs. study end) Student’s t-test, two-sided, using GraphPad Prism 6; *p < 0.05 vs. baseline.

Twenty patients were enrolled in the study. Participants’ characteristics at baseline and during the study are presented in Table 1 and in^22^. Even though patients randomized to ranolazine tended to present a lower systolic blood pressure in the course of the study, diastolic blood pressure was not different to control patients. Additionally, the smoker rate was higher (80% vs 20%) in the ranolazine group, whereas control patients more often presented hyperlipidaemia (70% vs 10%). We assessed plasma levels of important NO homeostasis markers. Baseline levels of arginine, homoarginine, ADMA and SDMA did not differ between groups (Fig. 1A–D). At the end of the study and compared to baseline, homoarginine levels had increased in the control group (Fig. 1A). This was not the case in ranolazine-patients. Interestingly, in ranolazine-treated-patients arginine plasma levels were significantly higher at the end of the study than at baseline (difference +26 µmol/L, 95% CI 8.6 to 44 µmol/L, Fig. 1B). ADMA and SDMA levels were not different.

**Table 1:** Patients‘ characteristics.

Characteristics	Standard (n = 10)	CI 95%	Ranolazine (n = 10)	CI 95%
Age (years)	66.8 ± 15.2	9.4	62.9 ± 14.1	8.8
Male sex	5.0 (50%)		6.0 (60%)	
Height	169.5 ± 6.5	4.0	171.2 ± 5.5	3.4
Weight	79.7 ± 19.6	12.2	75.2 ± 14.3	8.8
BMI	27.9 ± 8.2	5.1	25.5 ± 3.6	2.2
Arterial Hypertension	6.0 (60%)		5.0 (50%)	
Systolic BP Baseline	128.5 ± 16.2	10.1	117.2 ± 24.3	15.0
Diastolic BP Baseline	75.9 ± 10.5	6.5	73.0 ± 15.2	9.4
Systolic BP Week 6	139.1 ± 19.2	11.9	129.8 ± 28.9	17.9
Diastolic BP Week 6	81.8 ± 13.7	8.5	81.9 ± 14.0	8.7
Heart rate (beats/min) Baseline	75.0 ± 8.1	5.0	71.8 ± 8.1	5.0
Heart rate (beats/min) Week 6	71.8 ± 10.6	6.6	69.4 ± 10.6	6.6
Creatinine Baseline	0.9 ± 0.2	0.1	0.9 ± 0.2	0.1
Creatinine Week 6	1.0 ± 0.2	0.1	0.9 ± 0.2	0.1
GFR Baseline	91.8 ± 39.2	24.3	93.0 ± 19.7	12.2
GFR Week 6	83.1 ± 29.6	18.4	95.2 ± 40.3	25.0
Smoking	2.0 (20%)		8.0 (80%)$$	
Hyperlipidaemia	7.0 (70%)		1.0 (10%)$$	
Diabetes mellitus	2.0 (20%)		2.0 (20%)	
CAD Family History	1.0 (10%)		3.0 (30%)	
CAD vessel localisation: LAD	7.0 (70%)		5.0 (50%)	
CAD vessel localisation: CFX	4.0 (40%)		2.0 (20%)	
CAD vessel localisation: M1	0.0		3.0 (30%)	
CAD vessel localisation: RCA	1.0 (10%)		3.0 (30%)	

Data are given as mean ± SD and 95% confidence intervals (CI) or number and %. Comparisons were performed by paired (baseline vs study end) or unpaired (standard vs ranolazine) Student’s t-test, two-sided, using GraphPad Prism 6. ^$$^p < 0.01 vs standard.

Abbreviations used: BMI-Body Mass Index; BP-Blood pressure; min-Minute; GFP-Glomerular filtration rate; CAD-Coronary artery disease; LAD-Left anterior descending; CFX-Circumflex artery; M1-Marginal branch 1 of CFX; RCA-Right coronary artery.

At the start of the study, urinary excretion of 8-iso-PGF_2α_, was not significantly different between the groups (Fig. 1E). Even though there was no significant difference between baseline and values at the end of the study, 8-iso-PGF_2α_ concentrations showed a trend to lower values in ranolazine-patients (difference −144 pmol/mg creatinine, 95% CI −355 to 66, P = 0.15, Fig. 1E), whereas such a trend was missing in the control group.

## Discussion

Since ranolazine has been reported to improve myocardial blood flow in stable CAD patients^21^ we evaluated whether the application of ranolazine on top of guideline-based treatment has an impact on the arginine/NO pathway and urine 8-iso-PGF_2α_ in patients with a recent myocardial infarction. After 6 weeks of ranolazine, arginine plasma levels were significantly higher in ranolazine-treated patients. Even though no significant difference was obtained between baseline and at the end of the study, 8-iso-PGF_2a_ concentrations showed a trend to lower values in ranolazine-treated patients after 6 weeks, whereas 8-iso-PGF_2a_ concentrations between baseline and end of the study were not different in the control group. These findings support the hypothesis that ranolazine might improve diastolic blood flow without subsequent oxidative stress-induced NOS uncoupling, as previously shown for organic nitrates^10^.

Whether an augmentation in plasma arginine levels has beneficial effects remains controversial. Studies with oral arginine supplementation have produced both negative and positive results^11–14^. Schulman *et al*. reported that oral arginine supplementation did not have an effect on vascular function in STEMI patients^11^. Notably, arginine plasma levels after oral supplementation did not differ to levels in the placebo group. This is easily explainable by a high first pass clearance resulting in low arginine bioavailability^33^, which could explain the lack in clinical effect. Previous reports propose that the intravenous dose, but not the oral dose, is possibly associated with an increase in NO synthesis^34–36^. The mechanism for higher arginine levels after ranolazine treatment observed in our study remain elusive. Studies in precontracted rat aortic rings showed a concentration-dependent vasodilation in the presence of ranolazine, which could be reduced by inhibition of NO synthase^23^. It could be speculated that a ranolazine-induced increase in circulatory arginine promotes NO production by eNOS which in turn enhances vasodilation and improves myocardial blood flow. Additionally, ranolazine might suppress arginase activity. Neither homoarginine nor ADMA/SDMA are substrates of arginases in physiological concentration in contrast to arginine^37^. However, no data in literature exist and neither can our study contribute as to whether and/or how exactly ranolazine influences arginine homeostasis. But the ranolazine-mediated increase in arginine could be an additional NO/endothelium-dependent mechanism which could be beneficial in ACS.

Isoprostanes belong to a multifaceted family of compounds derived from arachidonic acid by lipid peroxidation^26,38,39^. It was reported that CAD patients with multi-vessel disease had higher levels of 8-isoprostane as patients with 1-vessel disease^40^ and that enhanced isoprostane formation predisposes patients to ACS^41^. In our study, 8-iso-PGF_2a_ had the tendency to be lower in the ranolazine treated group. It was previously reported that chronic administration of organic nitrates increases incident cardiovascular events in patients after myocardial infarction^42^. Of particular note, isosorbide-5-mononitrate has been reported to exert oxidative stress-mediated NOS uncoupling in experimental studies^10^. This might explain the unfavourable pharmacodynamics profile of organic nitrates in long-term treatment of ACS patients. In contrast, in our study ranolazine increased the substrate concentration of NOS, i.e. circulating arginine, by a yet unknown mechanism without subsequent increase in the oxidative stress marker 8-iso-PGF_2a_. Even more interesting, some data stress an important role of isoprostanes, in particular of 8-iso-PGF_2α_, in promoting atherosclerosis and vascular events as a mediator rather than as a marker^43,44^.

### Study limitations

Our study does not provide exact mechanisms on how ranolazine treatment influences arginine, homoarginine and ADMA/SDMA homeostasis. Since this pilot study was not adequately powered, we aim to initiate a larger study to fully evaluate the effect of ranolazine on NO homeostasis markers and isoprostane 8-iso-PGF_2α_ levels in patients with a recent myocardial infarction. Further research should also investigate possible mechanisms of ranolazine-induced arginine increase.

## Conclusion

In conclusion, our findings give evidence that ranolazine treatment enhances arginine plasma levels and lowers oxidative stress indicated by a trend to lower 8-iso-PGF_2α_ levels.

## Supplementary information

Supplemental Material
