# Supplementary material for: Effect of ranolazine on plasma arginine derivatives and urinary isoprostane 8-iso-PGF2α in patients with myocardial infarction in the randomized RIMINI-Trial
Source: Sci Rep. 2019 Apr 5;9:5708. doi: 10.1038/s41598-019-42239-1 (PMC6450888; doi:10.1038/s41598-019-42239-1)
Supplement: Supplementary file 1 — Supplemental Material [file 41598_2019_42239_MOESM1_ESM.pdf]

Effect of ranolazine on plasma arginine derivatives and urinary isoprostane 8-iso-PGF$_{2\alpha}$ in patients with myocardial infarction in the randomized RIMINI-Trial

Tjark F. Schwemer[4], Navina Deutscher[4], Nadine Diermann[4], Rainer Böger[1,2,3,5], Edzard Schwedhelm[1,2,3,5], Stefan Blankenberg[3,4], Felix W. Friedrich[1,2,3]*

Institutes of [1]Experimental and [5]Clinical Pharmacology and Toxicology, University Medical Center Hamburg-Eppendorf, Hamburg, Germany;

[2]Cardiovascular Research Center, University Medical Center Hamburg-Eppendorf, Hamburg, Germany;

[3]DZHK (German Centre for Cardiovascular Research), partner site Hamburg/Kiel/Lübeck, Germany;

[4]University Heart Center Hamburg, Hamburg, Germany;

*Correspondence to Felix W. Friedrich

Institute of Experimental Pharmacology and Toxicology, University Medical Center Hamburg-Eppendorf, Martinistraße 52, D-20246 Hamburg, Germany

Phone: +49-40-7410-53180; Fax: +49-40-7410-55925;

E-mail: f.friedrich@uke.de

Supplemental Material File, see also[1]

Methods

Patients and Study protocol

Twenty subjects between 35 and 60 years with unstable angina pectoris and proof of acute cardiac ischemia (serum levels of troponine-T-hs >53 pg/ml), angina pectoris >CCS II (Canadian Cardiovascular Society Classification of Angina Pectoris) and proof of myocardial dyskinesia entered the study. Main exclusion criteria were: refusal to participate in the study and/or parts of the study, younger than 18 years of age, acute cardio-pulmonary decompensation, middle and high grade liver insufficiency (Child-Pugh Score B and C), high grade renal insufficiency (creatinine-clearance <30 ml/min), homeless patients and drug-addicted patients, pregnant and/or breast-feeding women, treatment with ranolazine prior to enrolment, allergy against ranolazine, concomitant use of strong CYP3A4 inhibitors or of Class Ia and III antiarrhythmics (except amiodarone). A prior MI was no exclusion criteria. All patients included in the study received standard CAD treatment based on current guidelines[2]. For controlling and comparing of the ranolazine effect, the trial was performed in a two-armed, controlled, double-blind and randomized way achieved by a paper-based method. 10 patients received the study medication in addition to standard treatment and were tutored about the intake scheme. Ranolazine was given orally for 42 days, the first seven days 500 mg 2x/d, the next 35 days 750 mg ranolazine 2x/d. 10 patients received standard treatment without additional study medication.

References

1 Schwemer, T. F. *et al.* Effect of Ranolazine on Ischemic Myocardium IN Patients With Acute Cardiac Ischemia (RIMINI-Trial): A Randomized Controlled Pilot Trial. *Journal of cardiovascular pharmacology and therapeutics* **0**, 1074248418784290, doi:10.1177/1074248418784290 (2018).

2 Bassand, J. P. *et al.* [Guidelines for the diagnosis and treatment of non-ST-segment elevation acute coronary syndromes]. *Revista portuguesa de cardiologia : orgao oficial da Sociedade Portuguesa de Cardiologia = Portuguese journal of cardiology : an official journal of the Portuguese Society of Cardiology* **27**, 1063-1143 (2008).
